# MicroRNA-302a/d inhibits the self-renewal capability and cell cycle entry of liver cancer stem cells by targeting the E2F7/AKT axis

**DOI:** 10.1186/s13046-018-0927-8

**Published:** 2018-10-16

**Authors:** Yu-Shui Ma, Zhong-Wei Lv, Fei Yu, Zheng-Yan Chang, Xian-Ling Cong, Xiao-Ming Zhong, Gai-Xia Lu, Jian Zhu, Da Fu

**Affiliations:** 1 Central Laboratory for Medical Research, Shanghai Tenth People’s Hospital, Tongji University School of Medicine, Shanghai, 200072 China; 2 Department of Nuclear Medicine, Shanghai Tenth People’s Hospital, Tongji University School of Medicine, Shanghai, 200072 China; 3 Shanghai Engineering Research Center of Molecular Therapeutics and New Drug Development, College of Chemistry and Molecular Engineering, East China Normal University, Shanghai, 200062 China; 4 Department of Pathology, Shanghai Tenth People’s Hospital, Tongji University School of Medicine, Shanghai, 200072 China; 5 Department of Biobank, China-Japan Union Hospital, Jilin University, Changchun, 130033 China; 6 Department of Radiology, Jiangxi Provincial Tumor Hospital, Nanchang, 330029 China; 7 Department of Digestive Surgery, Rui Jin Hospital, Shanghai Jiao Tong University School of Medicine, Shanghai, 200025 China

**Keywords:** HCC, LCSCs, miRNA-302a/d, E2F7, Prognosis

## Abstract

**Background:**

There is increasing evidence that liver cancer stem cells (LCSCs) contribute to hepatocellular carcinoma (HCC) initiation and progression. MicroRNA (miRNA) plays a significant functional role by directly regulating respective targets in LCSCs-triggered HCC, however, little is known about the function of the miRNA-302 family in LCSCs.

**Methods:**

MiRNAs microarray was used to detect the miRNAs involved in LCSCs maintenance and differentiation. Biological roles and the molecular mechanism of miRNA-302a/d and its target gene *E2F7* were detected in HCC in vitro. The expression and correlation of miRNA-302a/d and E2F7 in HCC patients was evaluated by quantitative PCR and Kaplan–Meier survival analysis.

**Results:**

We found that the miRNA-302 family was downregulated during the spheroid formation of HCC cells and patients with lower miRNA-302a/d expression had shorter overall survival (OS) and progression-free survival (PFS). Moreover, E2F7 was confirmed to be directly targeted and inhibited by miRNA-302a/d. Furthermore, concomitant low expression of miRNA-302a/d and high expression of E2F7 correlated with a shorter median OS and PFS in HCC patients. Cellular functional analysis demonstrated that miRNA-302a/d negatively regulates self-renewal capability and cell cycle entry of liver cancer stem cells via suppression of its target gene *E2F7* and its downstream AKT/β-catenin/CCND1 signaling pathway.

**Conclusions:**

Our data provide the first evidence that E2F7 is a direct target of miRNA-302a/d and miRNA-302a/d inhibits the stemness of LCSCs and proliferation of HCC cells by targeting the E2F7/AKT/β-catenin/CCND1 signaling pathway.

**Electronic supplementary material:**

The online version of this article (10.1186/s13046-018-0927-8) contains supplementary material, which is available to authorized users.

## Background

Global morbidity of hepatobiliary malignancies, mainly hepatocellular carcinoma (HCC) and cholangiocarcinoma, has recently increased [1]. HCC has the third highest mortality rate out of all major malignant carcinomas worldwide [2]. Surgical resection, percutaneous ablation and liver transplantation are the standard treatments for HCC, however these are only applicable to a small proportion of patients with early disease [3]. Therefore, it is imperative to develop novel therapeutic strategies against HCC.

The high rate of recurrence and heterogeneity are the two major features of HCC [4]. Recent studies have suggested that liver cancer stem cells (LCSCs), which display the capacity for self renewal and differentiation, are located within the tumor bulk and are responsible for HCC initiation, progression, resistance to therapy and the hierarchical organization of tumor cells [5]. Moreover, based on the CSC theory, the presence of LCSCs may be of prognostic relevance in patients with HCC [6]. However, how LCSCs sustain their self-renewal remains largely unknown and the clinical significance of LCSCs is yet to be fully established.

MicroRNAs (miRNAs), a class of non-coding RNA molecules of 20–22 nucleotides, regulate the expression of target genes in a post-transcriptional manner. MiRNAs can effectively modulate various biological processes, including cell proliferation, migration, differentiation, and apoptosis [7–9]. Aberrant expression of miRNAs in a wide range of tumor types has been well established and known to functionally modulate various biological processes [10]. Accruing evidence has demonstrated that miRNAs play vital roles in regulating the self-renewal and tumorigenesis of CSCs in various types of cancer, including HCC [11–13]. Therefore, the dysregulation of specific miRNAs might serve as an effective biomarker for predicting prognosis in patients with cancer [14]. More importantly, previous studies have indicated that circulating miRNAs are stable enough to be detected in serum and plasma [15] and thus may be promising non-invasive biomarkers for the early detection and prognosis of cancers.

Because of the essential roles of LCSCs in initiation and recurrence, identification of key candidate miRNAs that regulate LCSCs and exploration of their therapeutic potential in HCC may be helpful for improving treatment [16]. To better identify the miRNAs involved in the LCSCs self-renewal and differentiation process, we analyzed the genome-wide transcriptional profiling with miRNA microarray in different stages of LCSC cells. We found that expression levels of several tumor-related miRNAs were altered during spheroid formation of HCC cell lines HepG2 and Huh7. Among these miRNAs, the miR-302 family was negatively correlated with stem cell phenotypes. We further demonstrated that miRNA-302a/d negatively regulated cell growth and spheroid formation, and promoted apoptosis of HCC cells via suppression of its target gene *E2F7*, which suggested that miRNA-302a/d and E2F7 might be potential tumor biomarkers for the diagnosis and treatment of HCC patients.

## Methods

### Ethics statement

The study was approved by the Ethics Committee of Shanghai Tenth People’s Hospital, Tongji University School of Medicine (SHSY-IEC-P-15-19). Each participant provided a written informed consent before participating in this study. All specimens were handled and made anonymous according to ethical and legal standards.

### Acquisition of clinical specimens

Fresh frozen tissue samples from HCC patients, who underwent surgical resection between 2008 and 2012, were obtained from the tissue bank of China-Japan Union Hospital and Shanghai Tenth People’s Hospital. These samples included paired tumor and adjacent non-cancerous tissues (*n* = 34), as well as a large cohort of individual HCC biopsies (*n* = 119) (Additional file 1: Table S1). The histological typing of these tumors and staging were performed according to the Seventh Edition of the American Joint Commission on Cancer (AJCC) tumor-node-metastasis (TNM) staging system for HCC [17], and patient data was collected up to December 31, 2017. The clinical information recorded included the patient’s characteristics (age and gender), tumor characteristics (tumor differentiation, diameter and number of foci), OS and DFS (Additional file 1: Table S1). The last follow-up was performed on May 30th 2018 by direct correspondence or phone interview. The occasion of mortality or tumor relapse was verified by patients or their relatives or from their medical records or the social security records. OS was analyzed for the months from the date of diagnosis to the time of mortality, regardless of the cause. DFS was defined as the period from the initial date of diagnosis to the time of tumor progression by computed tomography scan or to the time of mortality due to the disease.

### Cell lines

Human liver cancer lines HepG2 and Huh7 were purchased from the Cell Bank of the Chinese Academy of Sciences (Shanghai, China) and cultured in DMEM media (Invitrogen, Carlsbad, USA) and supplemented with 10% (*v*/v) fetal bovine serum (FBS), 100 U/ml penicillin, and 100 mg/ml streptomycin. Cell lines were routinely tested for mycoplasma contamination, and have been authenticated with short-tandem repeat analysis. Cell culture was conducted at 37 °C in a humidified 5% CO_2_ incubator.

### Cell proliferation assays

For the cell proliferation assays, 1,000 cells were seeded into 96-well plates to culture overnight. Next, CCK8 (10 L) reagent was then added to each well, the absorbance (A) was measured at 450 nm after 1 h, and the relative cell viability rate was calculated. All experiments were performed in triplicate.

### Cell cycle synchronization

For synchronization, cells were treated with 2 mM thymidine for 18–24 h, released for 8–10 h, then treated with thymidine for 16–18 h. S phase (about 4 h post-release) and mitosis were monitored by Hoechst 33342 (Beyotime, Jiangsu, China) staining. G2 cells (8 h post-release) were not incorporating Hoechst 33342, and the DNA did not condense. Mitotic cells were collected by shake-off once the cells showed an increase in the mitotic index (about 8–13 h post-release). The adherent cells were washed with PBS and then lysed. This population is not mitotic and is predominantly in G0. Isolated cells were replanted in a complete medium and incubated 4 h before harvesting for G1 cells [18].

### Tumor sphere assays

Cells were cultured as tumor spheres in DMEM containing 20 ng/mL hEGF (R&D Systems, Minneapolis, MN), 10 ng/mL hbFGF (R&D Systems), 4 mg/mL heparin sulfate (Sigma, St. Louis, MO), 0.15% bovine serum albumin (BSA) (Sigma), and 1% penicillin G-streptomycin [19]. Sphere forming capacity (SFC) was determined by the ability to form three-dimensional spheroids in culture over a period of three to seven days. For assessment of long term proliferation and fold expansion, spheres were disassociated and passaged every 7 days using 0.05% trypsin-EDTA (Invitrogen). Sphere cultures were grown in low-adherent flasks (Nunc, Penfield, NY). Cells were counted using a CountessTM (Invitrogen) automated cell counter. To induce tumor stem cell differentiation, the cultured tumorspheres of HepG2 and Huh7 cells were in medium with 10% (*v*/v) FBS for seven days at 37 °C in a humidified chamber supplemented with 5% CO_2_.

### Plasmid construction and transfection

Overexpression of hsa-miR-302a/d and E2F7 was performed in the pMSCV retroviral plasmid. The miR-302a/d sponge plasmid was constructed by inserting eight tandemly arrayed miR-302a/d-binding sites into the 3’UTR of dsRed [20]. All constructs were confirmed by sequencing. The plasmids were transiently transfected to target cells with lipofectamin 2000 (Invitrogen, cat.11668). To generate stable lines of overexpression or sponge knockdown, the plasmids were packaged into retrovirus with the amphotropic Phoenix packaging cell line and infected into target cells, followed by puromycin/hygromycin selection of the infected cells.

### Luciferase reporter assays

The human E2F7 3’-UTR oligonucleotides containing the wild-type (Wt) or mutant (Mut) miR-302a/d binding site were sub-cloned into the XhoI and NotI sites of the pGL3 luciferase reporter plasmid vector (Promega, Madison, WI). HeLa cells were seeded in 24-well plates and cultured for 24 h, then, cells were co-transfected with either the Wt or Mut reporter plasmid. Forty-eight hours after transfection, luciferase assay was determined using the Dual-Luciferase kit (Promega, Madison, WI) [21].

### RNA immunoprecipitation (RIP) assay

The EZ-Magna RIP Kit (Millipore, USA) was applied to conduct the RIP assay according to the product specification. Firstly, cells were collected and lysed in complete RIP lysis buffer. Then, the cell extract was incubated with RIP buffer containing magnetic beads conjugated to a human anti-Ago2 antibody (Millipore, USA). Samples were incubated with proteinase K with shaking to digest proteins and the immunoprecipitated RNA was isolated. Subsequently, the NanoDrop spectrophotometer was used to measure the concentration of RNA, and the purified RNA was subjected to real-time PCR analysis.

### RNA extraction and quantitative PCR (qPCR)

Total RNA, including miRNAs from normal liver tissue, HCC samples and cell lines, was isolated with Trizol reagent (Life Technologies, Grand Island, NY, USA). RNA concentration and purity were measured with the Nanodrop 1000 spectrophotometer (Thermo Fisher Scientific, Waltham, MA, USA) and 1.5% denaturing agarose gels, respectively. The cDNA was obtained from total RNA by reverse transcription, and the final RNA concentration used in the quantitative PCR reaction was 10 ng, qPCR was performed using the TaqMan universal PCR Kit (Life Technologies). The same amount of first-strand cDNA from each sample was used to detect the mRNA/miRNA expression levels using specific primers. Sequences of PCR primers are shown in the Additional file 1: Table S1. U6 and GAPDH were used as the endogenous controls, and the 2^-ΔΔCT^ method was used to analyze expression levels [22].

### Western blotting

Total protein from HepG2 and Huh7 cells was extracted using cell lysis buffer. Protein concentration was analyzed using standard procedures for Western blotting. After incubation with the appropriate horseradish peroxidase-conjugated secondary antibodies, the membranes were treated with an enhanced chemiluminescence reagent (Thermo Scientific, Dreieich, Germany), exposed to X-ray film (Kodak, Rochester, USA) and quantified by densitometry (Beckman, South Pasadena, Canada). The rabbit monoclonal antibody for CD133 (cat. ab216323), EpCAM (cat. ab32392), SOX2 (cat. ab92494), Nanog (cat. ab109250), AKT1 (cat. ab182729) and GAPDH antibody (cat. ab181602) were purchased from Abcam (Cambridge, UK) and used for western blot analyses. The rabbit polyclonal E2F7 antibody (Invitrogen, cat. PA5–68911) and goat anti-rabbit IgG (Merck) and goat anti-mouse IgG (Merck) antibodies were used for western blot analyses. Antibody dilutions were 1:1,000 for primary antibodies and 1:5,000 for secondary antibodies in western blotting.

### Flow cytometry analysis

Cell apoptosis was determined by flow cytometry analysis. Cells were collected, washed with cold PBS, fixed in cold 70% Ethanol, treated with DNase-free RNase (Sangon, RB473. 100 μg/ml) and stained with 50 μg/ml Propidium iodide (Sangon, cat. P1112) and Annexin V-APC/7-AAD kit (KeyGEN, cat. KGA-1025). The cells were analysed using a Gallios flow cytometer (Beckman Coulter) to quantify the proportion of cells in apoptosis status. Cells were harvested by trypsinization and counted such that about 1 × 10^6^ cells were used for the cell cycle analysis. The cells were washed in PBS, fixed in 70% ice-cold ethanol overnight at 4 °C, and then washed in PBS and incubated in 1 mL staining solution (20 mg/mL propidium iodide, 10 U/mL RNaseA) for 30 min at room temperature. The DNA content was measured by flow cytometry on a FACS Calibur system (Becton Dickinson), and the cell cycle distributions of the different populations were determined using “Flowjo” software (Verity Software House).

### Subcutaneous mouse xenografts

A total of 80 male BALB/c nude mice (15–18 g), aged 4–5 weeks old, were purchased from Shanghai SLAC Laboratory Animal Co., Ltd., (Shanghai, China). Mice were equally divided into two groups (*n* = 6) and received a subcutaneous injection in their right flank of either 1 × 10^7^ HepG2 and Huh7 cells stably expressing miR-302a/d and/or E2F7. Mice were housed in an animal facility at a temperature of 25 °C and humidity of 60–70% and subjected to a 12 h light-dark cycle with ad libitum access to food and water. Tumor growth was monitored twice a week by size measurement. Both maximum (L) and minimum (W) diameters of the tumors were measured using a slide caliper, and the tumor volume was calculated as πLW^2^/6 [23].

### Microarray analysis

For microarray analysis of miRNA gene expression, Agilent Array platform was employed. The sample preparation and microarray hybridization were performed based on the manufacturer’s standard protocols. Briefly, 1 μg of total RNA from each sample was amplified and transcribed into fluorescent cDNA with using the manufacturer’s Agilent’s Quick Amp Labeling protocol (version 5.7, Agilent Technologies). Agilent Quick Amp Labeling Kit was used for sample labeling. Hybridization was performed in Agilent’s SureHyb Hybridization Chambers. The labeled cRNAs were hybridized onto the Whole Human Genome Oligo Microarray (4 × 44 K, Agilent Technologies). After having washed the slides, the arrays were scanned by the Agilent Scanner G2505B. Agilent Feature Extraction software (version 11.0.1.1) was used to analyze the acquired array images. Quantile normalization and subsequent data processing were performed using the GeneSpring GX v11.5.1 software package (Agilent Technologies).

### Bioinformatics analysis

Hierarchical clustering was performed using the multiple experiment viewer (MeV) 4.7.1 software programs: (http://www.tm4.org/mev/). We used four online target-gene prediction software miRDB (http://mirdb.org/miRDB/), miRTarBase (http://mirtarbase.mbc.nctu.edu.tw/), miRanda (www.microrna.org) and RNA22 (http://cbcsrv.watson.ibm.com/rna22.html) to forecast several potential target genes of hsa-miR-302a/d. Subsequently, predicted target genes were subjected to KEGG pathway online analysis by DAVID (http://david.ncifcrf.gov).

### Statistical analysis

Data is presented as mean ± standard deviation. The χ^2^ was used to compare the differences of categorical variables and the Student’s t-test was used for comparison of differences between two groups. Kaplan-Meier curves and the log-rank test were used to analyze the OS or DFS of HCC patients. All statistical analyses were performed using the SPSS 20.0 software program (SPSS Inc., Chicago, IL, USA). A *P*-value < 0.05 was considered statistically significant.

## Results

### In vitro formation and identification of tumor spheres from HCC cell lines

**Fig. 1:**
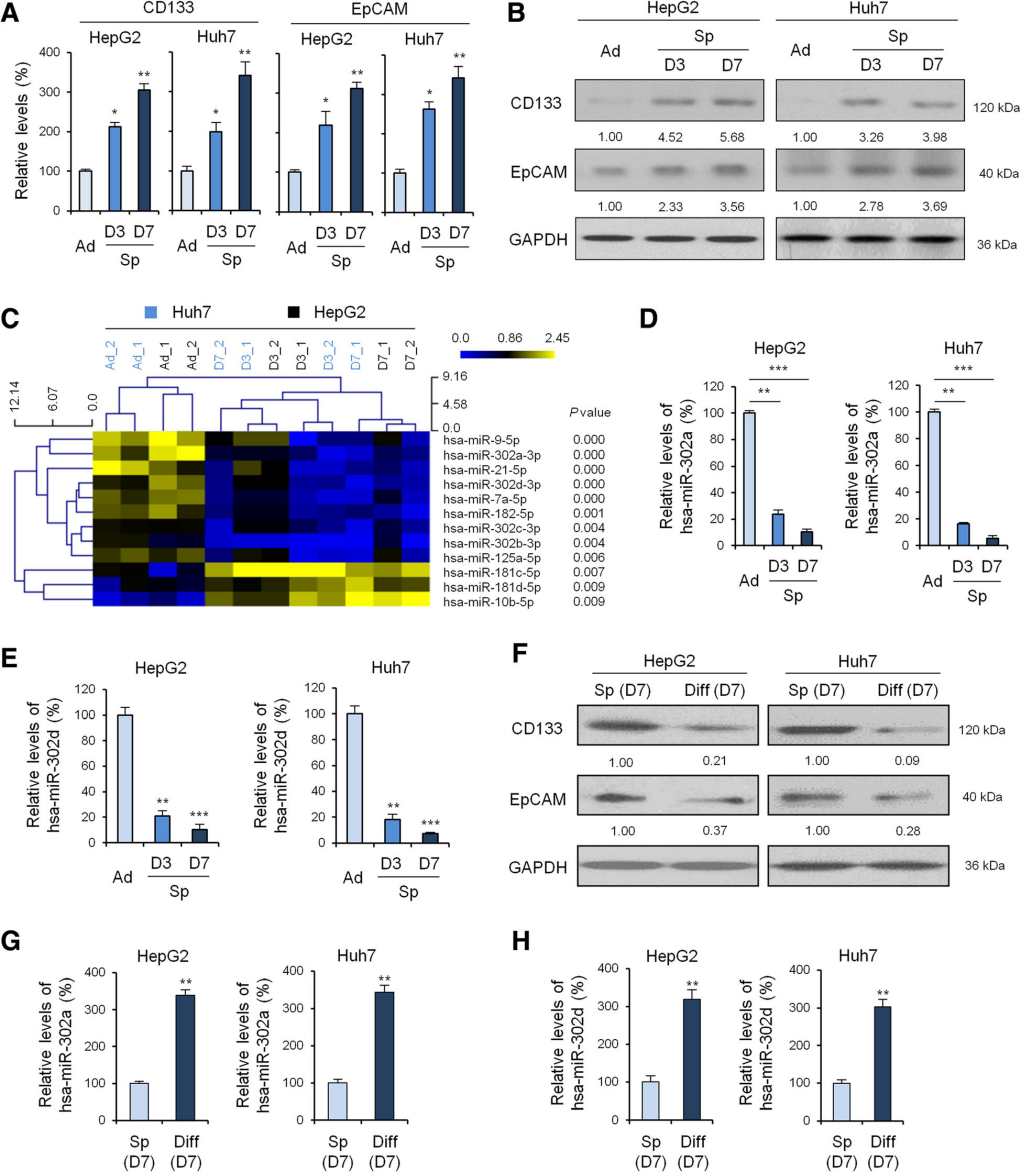
Expression of CSC markers and miRNA-302 family in LCSCs. qRT-PCR (**a**) and Western blot (**b**) to quantify CD133 and EpCAM level in adherent and tumor spheres of HepG2 and Huh7 cells after 3 days cultured in stem cell medium containing EGF and bFGF. GAPDH was used as loading control. **c**, MiRNAs microarray analysis to identify key miRNA molecules implicated in LCSCs maintenance. qRT-PCR to validate the miRNA-302a (**d**) and -302d (**e**) level in adherent and tumor spheres of HepG2 and Huh7 cells after 3 days cultured in stem cell medium containing EGF and bFGF. **f**, Western blot to quantify CD133 and EpCAM level in tumor spheres of HepG2 and Huh7 cells at Day 7 cultured in stem cell medium and differentiated liver cancer cells. qRT-PCR to quantify the miRNA-302a (**g**) and -302d (**h**) mRNA level in adherent and tumor spheres of HepG2 and Huh7 cells at Day 7 cultured in stem cell medium and differentiated liver cancer cells. Data shown are the means ± SD of three independent experiments. Statistical analyses were performed with one-way ANOVA (*P < 0.05, **P < 0.01, and ***P < 0.001 vs. normal)

HCC cell lines have distinct phenotypes and fall into well or poorly differentiated clusters [24, 25]. In this study, to obtain tumor spheres from HCC cell lines in vitro, two well-differentiated hepatocyte-derived carcinoma cell lines HepG2 and Huh7 cells were chosen and cultured as adherent monolayers. Both cell lines exhibited low expression of several CSC markers (Fig. 1a, b and Additional file 1: Figure S1A, B), thus likely to portray stem cell features. Cells were then transferred to stem cell medium containing EGF and bFGF. After 3 days in culture macroscopic and spherical or oval tumor spheres formed. Continuing proliferation led to a progressive 5- to 10-fold increase in the diameter of the tumor spheres over 7 days. The identification of CSC remains challenging and several CSC markers have been proposed. Among these marker, CD133 and EpCAM expression seems to better correlate the presence of LCSC and aggressiveness of liver tumors [26]. Our results showed that the cultured tumorigenic liver tumor spheres exhibited stem-like features with gradual and significantly increased expression of CD133 and EpCAM on the both mRNA (Fig. 1a) and protein (Fig. 1b) expression level.

Next, the expression level of stem cell markers connected to self-renewal and stemness capability was evaluated. To this end, we examined the gene expression of Sox2 and Nanog transcripts in samples extracted from adherent vs. spheroids of HepG2 and Huh7 cells. As shown in Additional file 1: Figure S1A, the expression of *Sox2* and *Nanog* were significantly increased in cell grown as spheres compared with those grown in adherence. The protein expression of Sox2 and Nanog was also significantly increased in cells grown as spheres compared with those grown in adherence (Additional file 1: Figure S1B).

### MiRNA microarray analysis reveals that the miRNA-302 family is involved in LCSCs maintenance

To identify key miRNA molecules and mechanisms implicated in LCSCs maintenance, we profiled the expression of miRNAs in these two HCC cell types. When compared with the expression levels of miRNAs from both sample groups, our results showed that the gene expression level of miRNA-302 family was gradually and significantly decreased during formation of tumor spheres from HepG2 and Huh7 cells (Fig. 1c).

To validate these differentially regulated miRNAs, we examined the expression of miRNA-302a, −302b, −302c, and -302d in RNA extracted from adherent vs. spheroids of HepG2 and Huh7 cells. As shown in Fig. 1d-e and Additional file 1: Figure S1C-D, the expression of the indicated transcripts, analyzed by real-time RT-PCR, were significantly decreased in cells grown as spheres compared with those grown in adherence. Among them, the expression levels of miRNA-302a and -302d in tumor spheres from HepG2 and Huh7 cells were significantly decreased when compared with the control group (*P* < 0.001) (Fig. 1d, e).

### Differentiation and CSC markers expression of LCSCs

Tumor spheres cultured in suspension may redifferentiate to adherent cells when cultured in routine medium containing serum. Therefore, we detected CSC marker expression in tumor spheres from HepG2 cells cultured in suspension with serum-free medium to maintain stemness or in monolayers with the addition of FBS to induce differentiation. Our data showed that the gene levels (Additional file 1: Figure S2A) and protein expression (Fig. 1f) of liver stem cell-associated genes (including CD133 and EpCAM) in re- differentiated cells were significantly reduced following FBS treatment. Moreover, the gene levels (Additional file 1: Figure SB) and protein expression (Additional file 1: Figure S2C) of stem cell-associated genes (including Sox2 and Nanog) in re- differentiated cells were also significantly reduced following FBS treatment. Conversely, the expression of miRNA-302 family was significantly increased in adherent HepG2 and Huh7 cells when compared with tumor spheres cultured in suspension (Fig. 1g-h and Additional file 1: Figure S2D-E).

To exclude the possible effects of serum, expression levels of miRNA-302a and -302d mRNA in HepG2 and Huh7 cells were investigated after culture in DMEM supplemented with either 5, 10 or 15% FBS. The qPCR analyses revealed that changes in serum concentration had no effect on the expression levels of miRNA-302a (Additional file 1: Figure S2F) and -302d (Additional file 1: Figure S2G), indicating that the observed changes in miRNA-302a and -302d expressions were associated with stemness and not serum concentration. Moreover, to exclude the possible effects of additional growth factors, expression levels of miRNA-302a and -302d mRNA in HepG2 and Huh7 cells were investigated after culture in DMEM supplemented with or without additional growth factors. The qPCR analyses revealed that additional growth factors had no effect on the expression levels of miRNA-302a and -302d (Additional file 1: Figure S2H), indicating that the observed changes in miRNA-302a and -302d expressions were associated with stemness and not additional growth factors.

### MiRNA-302a/d inhibits proliferation and spheroid formation and promotes cellular apoptosis in HCC cells

**Fig. 2:**
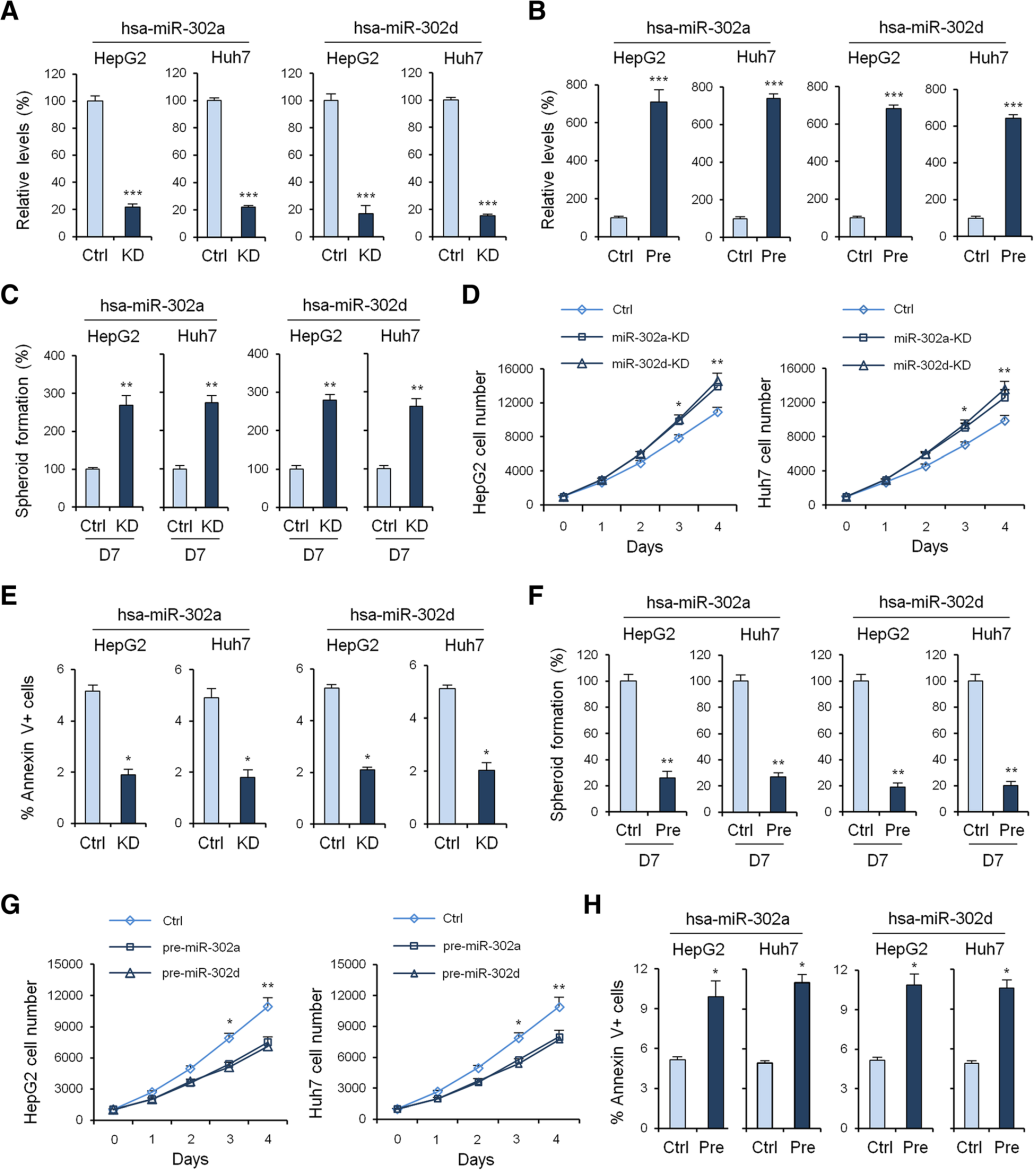
Biological role of miRNA-302a/d in HCC in vitro. qRT-PCR measurement of the levels of miRNA-302a/d mRNA in HepG2 and Huh7 cells before and after knockdown (**a**) or overexpression (**b**) of miRNA-302a/d. Data shown are the means ± SD of three independent experiments. **c**, Tumor sphere assays of HepG2 and Huh7 cells before and after knock down of miRNA-302a/d. **d**, HepG2 and Huh7 cell counts in 96-well plate after transfection with negative control or miRNA-302a/d knockdown plasmids at the indicated day. **e**, HepG2 and Huh7 cell apoptosis was examined by flow cytometry analysis before and after knockdown of miRNA-302a/d. **f**, Tumor sphere assays of HepG2 and Huh7 cells before and after overexpression of miRNA-302a/d. **g**, HepG2 and Huh7 cell counts in 96-well plate after transfection with negative control or miRNA-302a/d overexpression plasmids at the indicated day. **h**, HepG2 and Huh7 cell apoptosis was examined by flow cytometry analysis before and after overexpression of miRNA-302a/d. Statistical analyses were performed with one-way ANOVA (*P < 0.05, **P < 0.01, and ***P < 0.001 vs. normal)

To investigate the biological role of miRNA-302a/d in HCC cells, we performed loss- and gain-of-function studies using sponge (Fig. 2a) and overexpressed plasmid (Fig. 2b) in HepG2 and Huh7 cells. As shown in Fig. 2c and d, suppression of miRNA-302a/d significantly enhanced the spheroid formation and growth rate of HepG2 and Huh7 cells transfected with the miRNA-302a/d sponge plasmid compared with the negative control-transfected cells. Moreover, suppression of miRNA-302a/d significantly decreased the cellular apoptosis of HepG2 and Huh7 cells transfected with the miRNA-302a/d sponge plasmid compared with the negative control- transfected cells (Fig. 2e). However, spheroid formation and cellular growth assay revealed that following miRNA-302a/d overexpression, the spheroid formation (Fig. 2f) and growth rate (Fig. 2g) of HepG2 and Huh7 cells were significantly inhibited when compared to the control group. Cellular apoptosis assay revealed that following miRNA-302a/d overexpression, the cellular apoptosis of HepG2 and Huh7 cells were significantly increased when compared to the control group (Fig. 2h).

### MiR-302a/d directly targets E2F7 in human HCC cells

Although the large majority of miRNA targets remain unknown, there is evidence for redundant target specificity of unrelated miRNAs, or miRNAs from the same family [27]. Next, we searched for potential target genes of miR-302a/d using four publicly available databases including miRanda, RNA22, miRDB and miRTarBase to predict the potential target genes of miRNA-302 family.

**Fig. 3:**
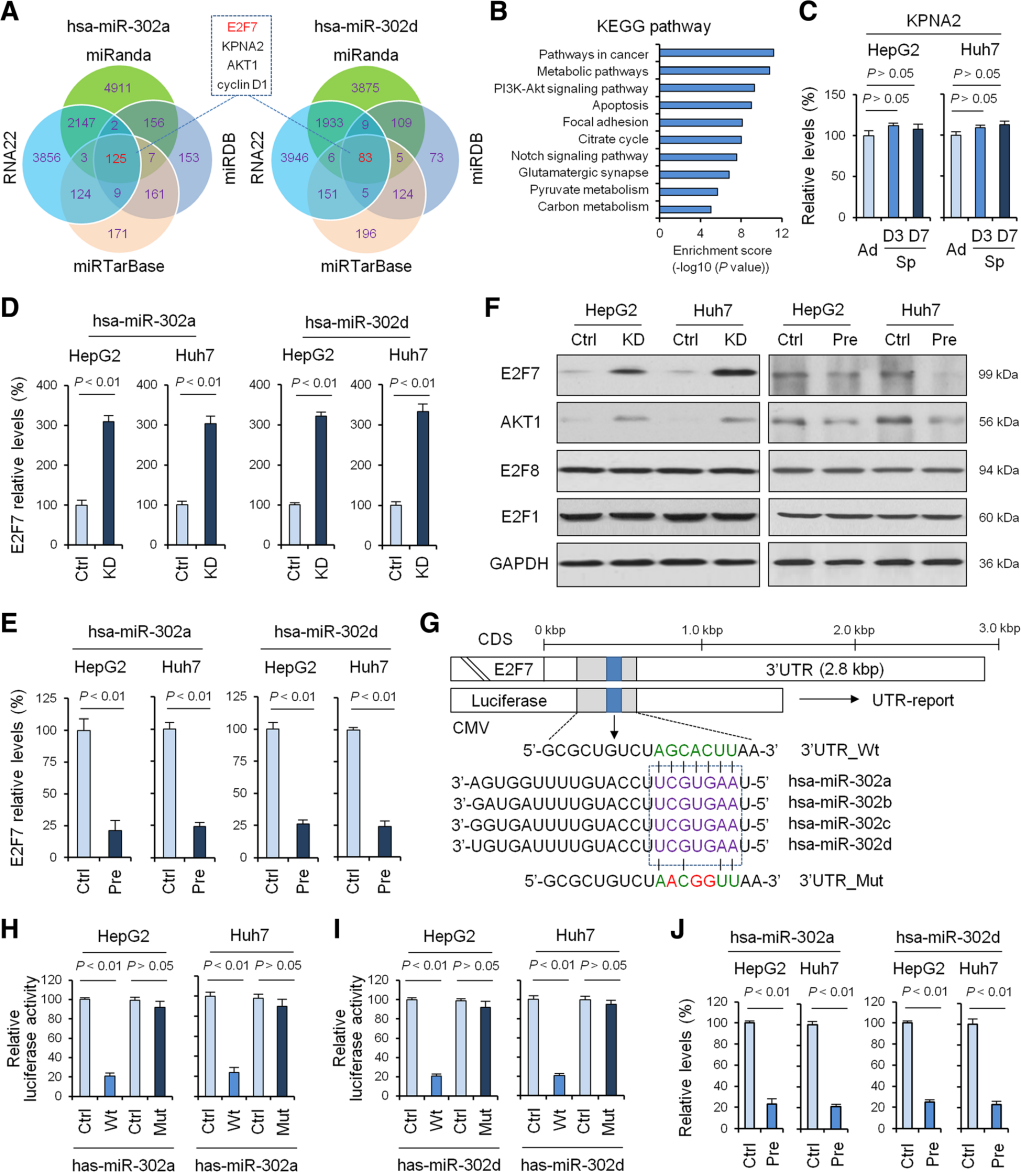
Validation of E2F7 as a direct target of miRNA-302a/d. **a**, miRNA-302a and miRNA-302d target prediction using four target genes prediction programs. **b**, KEGG analysis of 208 common predicted target genes. **c**, qRT-PCR to quantify KPNA2 level in adherent and tumor spheres of HepG2 and Huh7 cells after 3 days cultured in stem cell medium containing EGF and bFGF. qRT-PCR was used to measure the mRNA level of E2F7 after treatment of miRNA-302a/d knockdown (**d**) or overexpression (**e**). **f**, Western blotting was used to measure the protein levels of potential target genes after treatment of miRNA-302a/d knockdown or overexpression. AKT1 was used as a positive control for miRNA-302a/d target. **g**, A schematic diagram of the miRNA-302 family of 3 ‘UTR in E2F7 binding site and mutation site. Luciferase activity assay of pGL3-E2F7–3’UTR reporter co-transfected with miRNA-302a (**h**) and -302d (**i**) mimic or mutational oligonucleotides in HepG2 and Huh7 cells. **j**, RIP-IP assays were performed to co-IP the Ago2 complexes from HepG2 and Huh7 cells transfected with either hsa-miR-302a/d mimic or negative control mimic. Real-time PCR assays were performed on RNA samples isolated from the Ago2 co-IP fractions to measure the relative enrichment of the E2F7 mRNA. Data shown are the means ± SD of three independent experiments. Statistical analyses were performed with one-way ANOVA (** = *P* < 0.01, *** = *P* < 0.001)

The result showed that there were 125 and 83 common predicted target genes for miR-302a and -302d (Fig. 3a), respectively. Of these, 4 common genes were predicted as the targets for miR-302a and -302d, including 2 reported genes (*AKT1* [28] and *cyclin D1* [29]) and 2 previously unreported potential target genes (*E2F7* and *KPNA2*) (Fig. 3b). Subsequently, predicted target genes were subjected to KEGG pathway analysis and the results revealed some important cancer and stem-related pathways including metabolic pathways, apoptosis, pathways in cancer, focal adhesion, Notch and PI3K-Akt signaling pathway (Fig. 3c), in which the transcription factor E2F7 was a candidate involved in tumorigenesis and cancer stemness in HCC. Moreover, the mRNA level of KPNA2 was undifferentiated in cells grown as spheres compared with those grown in adherence (Fig. 3c). Thus, in our current study, we were particularly interested in E2F7 expression and its correlation with miR-302a and -302d.

The expression of E2F7 is associated with cell cycle and E2F7 RNA and protein levels oscillate through the cell cycle with the highest expression levels during S-phase [30]. Therefore, we detected E2F7 mRNA level after treatment of miR-302a/d knockdown or overexpression in synchronized HepG2 and Huh7 cells to determine whether miR-302a/d really altered the expression levels of E2F7 independent of its effects on cellular proliferation. When compared to the control group, the gene expression of E2F7 was significantly higher in miRNA-302a/d knockdown group (*P* < 0.01) (Fig. 3d, Additional file 1: Figure S3A-B) and was significantly lower in the miRNA-302a/d overexpression group (*P* < 0.01) (Fig. 3e), which suggested that E2F7 was regulated by miRNA-302a/d and was negatively correlated with miRNA-302a/d.

Next, to further test the regulation role of miRNA-302a/d on E2F7 at the protein level, we used Western blot analysis to measure the protein levels of E2Fs family members (E2F1, E2F7, and E2F8) and AKT1, a reported downstream protein after miRNA-302a/d overexpression or knockdown. The results suggested that the increase in miRNA-302a/d levels significantly decreased E2F7 protein expression and had the same tendency in AKT1 and vice versa (Fig. 3f). However, there was no obvious change of the protein levels of E2F8, another atypical E2F family member, and E2F1, which participates in the self-renewal and differentiation of stem cells, after miRNA-302a/d overexpression or knockdown (Fig. 3f).

Next, by using bioinformatics analysis, we found that miRNA-302 family contained specific binding sequence of the 3’-UTR region of E2F7 gene. The 3’-UTR binding site and mutation site of miRNA-302a/d of E2F7 gene are shown in Fig. 3g. We performed a luciferase reporter assay to further verify whether miRNA-302a/d directly targeted E2F7. As shown in Fig. 3h and i, ectopic expression of miRNA-302a/d decreased the luciferase activity of the 3’-UTRs of E2F7. However, miRNA-302a/d mutant containing three altered nucleotides in the seed sequence did not have an inhibitory effect on luciferase activity.

To further confirm that hsa-miR-302a/d directly interacts with E2F7 mRNA, we tested hsa-miR-302a/d-mediated binding of RISC to E2F7 mRNA using the Ago2-based ribonucleoprotein-IP assay (RIP-IP). One irrelevant/binding miRNA was used as a negative control. As shown in (Fig. 3j), the Ago2 co-IP fraction from cells treated with the hsa-miR-302a/d mimic was significantly enriched for E2F7 mRNA compared to cells treated with control miRNA mimics.

### Expression of E2F7 in formation and differentiation of LCSCs

**Fig. 4:**
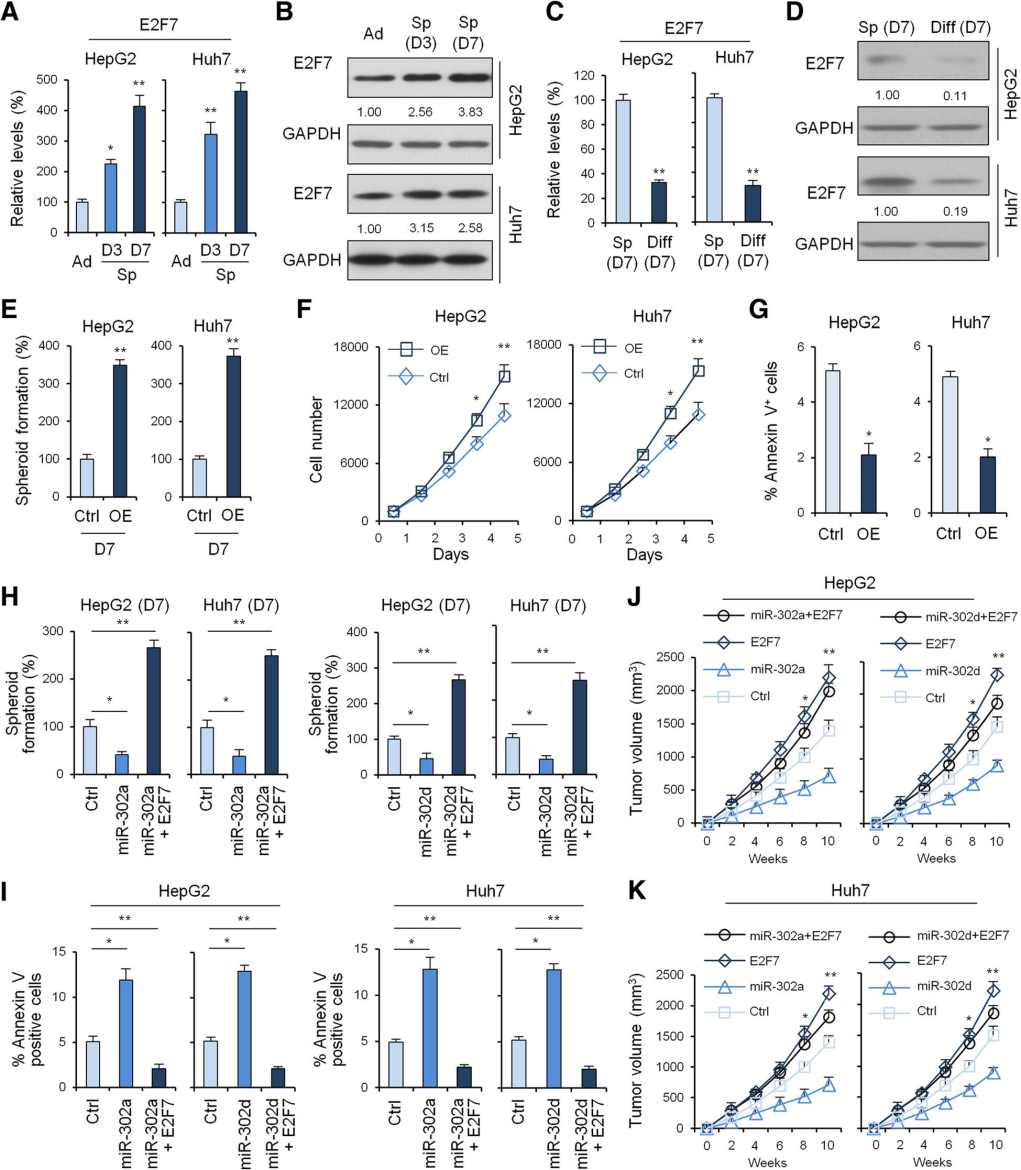
Biological role of miRNA-302a/d and E2F7 in HCC in vitro and in vivo. qRT-PCR (**a**) and Western blot (**b**) to quantify E2F7 levels in adherent and tumor spheres of HepG2 and Huh7 cells after 3 and 7 days cultured in stem cell medium containing EGF and bFGF. GAPDH was used as loading control. qRT-PCR (**c**) and Western blot (**d**) to quantify E2F7 levels in tumor spheres of HepG2 and Huh7 cells at Day 7 cultured in stem cell medium and differentiated liver cancer cells. **e**, Tumor sphere Assays of HepG2 and Huh7 cells before and after overexpression of E2F7. **f**, HepG2 and Huh7 cell counts in 96-well plate after transfection with negative control and E2F7 overexpression plasmids at the indicated day. **g**, HepG2 and Huh7 cell apoptosis was examined by flow cytometry analysis before and after overexpression of E2F7. Statistical analyses were performed with one-way ANOVA (*P < 0.05, **P < 0.01, and ***P < 0.001 vs. normal). **h**, Tumor sphere assays of HepG2 and Huh7 cells before and after overexpression of miRNA-302a/d and/or E2F7. **i**, HepG2 and Huh7 cell apoptosis was examined by flow cytometry analysis before and after overexpression of miRNA-302a/d and/or E2F7. The subcutaneous tumor capacity of HepG2 and Huh7 cells before and after overexpression of miRNA-302a (**j**) and miRNA-302d (**k**) and/or E2F7 was detected. Statistical analyses were performed with one-way ANOVA (*P < 0.05, **P < 0.01, and ***P < 0.001 vs. normal)

Next, we analyzed the gene expression of E2F7 in formation and differentiation of LCSCs to explore the association between liver cancer stemness and E2F7 level. The results showed that the gene expression (Fig. 4a) and protein level (Fig. 4b) of E2F7 was gradually and significantly increased during formation of tumor spheres in HepG2 and Huh7 cells. Moreover, when analyzing the correlation of miRNA-302a/d and E2F7 gene in adherent vs. spheroids of HepG2 and Huh7 cells, we found that the relative expression level between miRNA-302a/d and E2F7 presented an inverse correlation (Additional file 1: Figure S3C, D).

Instead, the gene levels (Fig. 4c) and protein expression (Fig. 4d) of E2F7 in re-differentiated cells were significantly reduced following FBS treatment and the gene level of E2F7 was negatively correlated with miRNA-302a or miRNA-302d in adherent HepG2 and Huh7 cells when compared with tumor spheres cultured in suspension (Additional file 1: Figure S3E, F).

### Biological role of E2F7 in HCC in vitro

To investigate the biological role of E2F7 in HCC, we performed gain-of-function studies using E2F7 overexpression on HepG2 and Huh7 cells (Additional file 1: Figure S4A, B). As shown in Fig. 4e and f, overexpression of E2F7 significantly enhanced the spheroid formation and growth rate of HepG2 and Huh7 cells transfected with the E2F7 overexpression plasmid compared with the negative control-transfected cells. Moreover, overexpression of E2F7 significantly decreased the cellular apoptosis of HepG2 and Huh7 cells transfected with the E2F7 overexpression plasmid compared with the negative control-transfected cells (Fig. 4g). These results indicated that E2F7 may be involved in stemness and proliferation in HCC in vitro.

Next, we stably overexpressed both miRNA-302a/d and E2F7 in HepG2 and Huh7 cells (Additional file 1: Figure S4C-F). As expected, E2F7 overexpression abolished the effects of miRNA-302a/d in liver cancer cell spheroid formation (Fig. 4h). Similarly, with forced expression of E2F7, miRNA-302a/d was incapable of suppressing the cancer cell growth (Additional file 1: Figure S4G, H). Furthermore, cellular apoptosis was reduced in HepG2 and Huh7 cells when miRNA-302a/d and E2F7 were both overexpressed (Fig. 4i). Importantly, upon evaluating the effect of overexpression of both miRNA-302a/d and E2F7 on the model of HepG2 and Huh7 tumor cells subcutaneously implanted in nude mice, our results showed that overexpression of E2F7 abolished the effects of miRNA-302a (Fig. 4j) and miRNA-302d (Fig. 4k). Hence, our results showed that in the presence of E2F7 overexpression, miRNA-302a/d failed to inhibit proliferation of liver cancer cells in vitro and in vivo, which suggested that miRNA-302a/d inhibits cancer cell stemness and proliferation and promotes liver cancer cell apoptosis through suppressing E2F7.

### MiRNA-302a/d inhibits the spheroid formation of LCSCs through restrained cell cycle entry

**Fig. 5:**
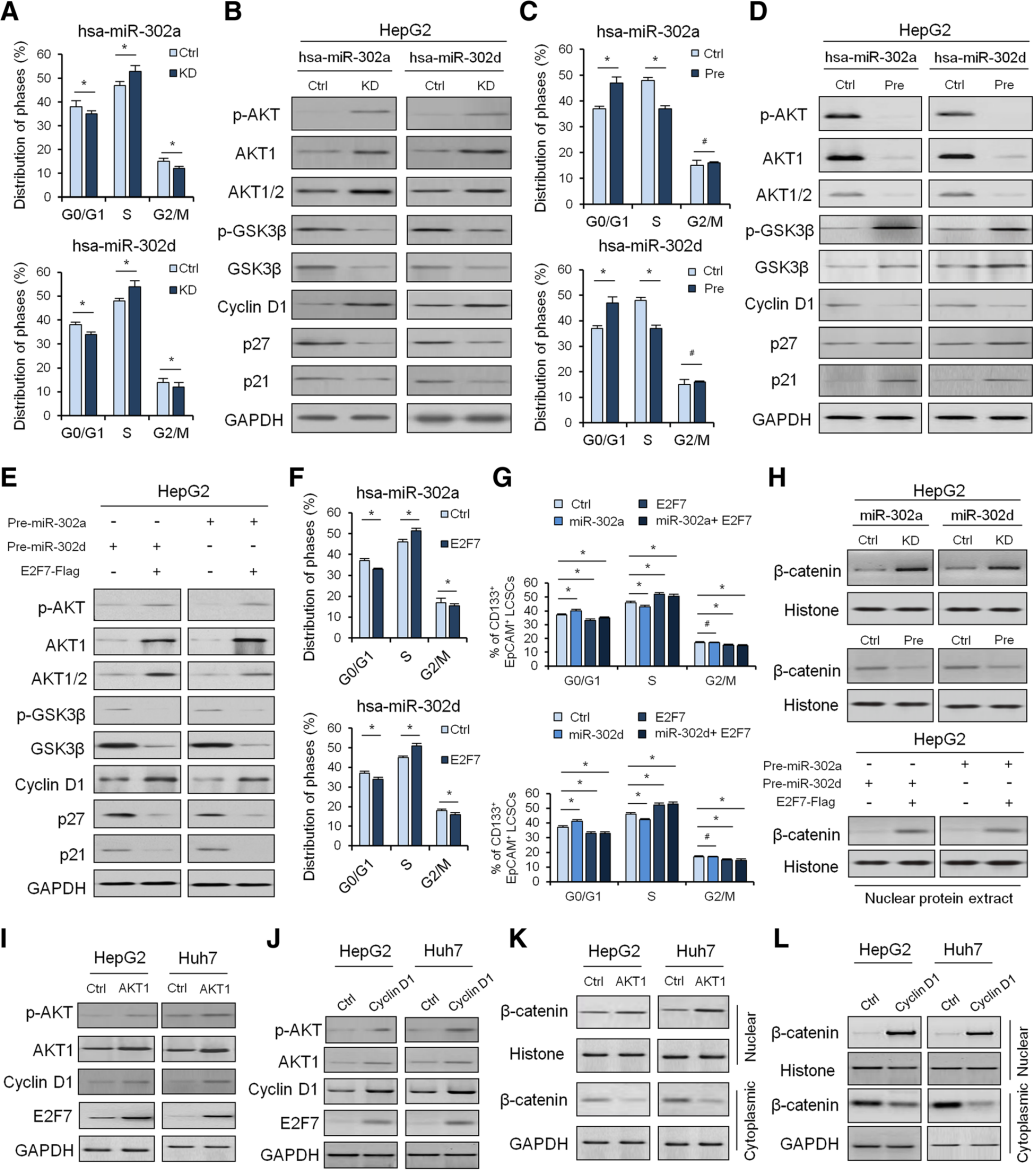
The effect of miRNA-302a/d and/or E2F7 on AKT1-cyclin D1 signaling and downstream cell cycle. **a**, Cell cycle quantitative analysis of HepG2 cells transfected with the miRNA-302a/d knockdown or the control vector. **b**, Western blot to quantify the effect of miRNA-302a/d knockdown on AKT1-cyclin D1 signaling in HepG2 cells. **c**, Cell cycle quantitative analysis of HepG2 cells transfected with the miRNA-302a/d overexpression or the control vector. **d**, Western blot to quantify the effect of miRNA-302a/d overexpression on AKT1-cyclin D1 signaling in HepG2 cells. **e**, Western blot to quantify the effect of miRNA-302a/d and E2F7 overexpression on AKT1-cyclin D1 signaling in HepG2 cells. **f**, Cell cycle quantitative analysis of HepG2 cells transfected with the miRNA-302a/d and E2F7 overexpression or the control vector. **g**, Cell cycle quantitative analysis of CD133^+^EpCAM^+^ HepG2 cells transfected with the miRNA-302a/d and/or E2F7 overexpression or the control vector. **h**, Western blot to quantify the effect of miRNA-302a/d and/or E2F7 on protein levels of intranuclear β-catenin level in HepG2 cells. Western blot to quantify the effect of AKT1 (**i**) and Cyclin D1 (**j**) on E2F7 protein level in HepG2 and Huh7 cells. Western blot to quantify the effect of AKT1 (**k**) and Cyclin D1 (**l**) on intranuclear β-catenin level in HepG2 and Huh7 cells

Knockdown of miR-302a/d led to significant G1 arrest or delayed the entry of S phase in HepG2 and Huh7 cells as compared with control (Fig. 5a), suggesting that miR-302a/d inhibits cell proliferation and cell cycle entry through the AKT1-p27^Kip1^/p21^Cip1^ pathway.

To further understand how miRNA-302a and -302d inhibits the spheroid formation of LCSCs, the levels of cell cycle regulatory pathway protein AKT1 and its downstream effectors were analyzed by Western blotting. Levels of both total AKT1 and phosphorylated AKT (p-AKT) were enhanced in miR-302a/d-knock down HepG2 and Huh7 cells compared to the corresponding control cells (Fig. 5b). Expression of AKT’s downstream effector, cyclin D1, was also elevated. However, expression of total GSK3β and phosphorylated GSK3β, cyclin-dependent kinase (CDK) inhibitors p27^Kip1^ and p21^Cip1^, was down-regulated (Fig. 5b). On the contrary, overexpression of miR-302a/d led to significant G1 entry in HepG2 and Huh7 cells as compared with control (Fig. 5c). Moreover, with miR-302a/d overexpression, levels of total AKT1 and p-AKT were notably reduced as expected, as well as cyclin D1 (Fig. 5d). Inversely, total GSK3β and phosphorylated GSK3β, and cell cycle inhibitors p27^Kip1^ and p21^Cip1^ expression were elevated accordingly after HepG2 and Huh7 cells were transfected with miR-302a/d precursors or control.

### E2F7 activates AKT1-cyclin D1 signaling and the downstream cell cycle

In order to illustrate whether the observed downregulation of E2F7 during overexpression of miR-302a/d was related to the reduced proliferation rate, we overexpressed the E2F7 lacking the 3’-UTR region (E2F7 CDS) into miR-302a/d-transfected HepG2 cells to determine the role of E2F7 on AKT1 signaling and downstream cell cycle. Interestingly, we found that E2F7 not only enhanced levels of phospho-Akt, but also significantly increased the expression of cell cycle related gene cyclin D1. In addition, total GSK3β and phosphorylated GSK3β, p21, and p27 were downregulated after E2F7 overexpression (Fig. 5e). Overexpression of E2F7 led to significant G1 entry or promoted the entry of S phase in HepG2 and Huh7 cells as compared with control (Fig. 5f). Moreover, overexpression of E2F7 in miR-302a/d-transfected HepG2 cells led to significant G1 entry or promoted the entry of S phase in CD133^+^/EpCAM1^+^ hepatoma cells as compared with control (Fig. 5f), suggesting that E2F7 promotes cell cycle entry through the AKT1-p27^Kip1^/p21^Cip1^ pathway independent of miR-302a/d.

### MiR-302a/d collaborates with E2F7 to regulate β-catenin/CCND1 signaling

Cyclin D1, a major downstream target gene of Wnt signaling, was remarkably decreased in miR-302a/d overexpression cells (Fig.5c), indicating that the Wnt pathway may be involved in miR-302a/d and E2F7 functions during spheroid formation and cell cycle entry of LCSCs. We further examined the protein level of the key upstream regulator, β-catenin, in HCC cells with miR-302a/d knockdown and/or E2F7 overexpression by Western blot analysis, which showed that the levels of intranuclear β-catenin were strikingly enhanced (Fig. 5h). On the contrary, levels of intranuclear β-catenin were reduced in pre-miR-302a/d-transfected HepG2 cells compared to the corresponding control cells (Fig. 5h).

### Feedforward regulatory E2F7 signaling by AKT/CCND1 axis

Since E2F7 is known to be highly expressed in proliferating cells and its RNA and protein levels oscillate through the cell cycle with the highest expression levels during S-phase [31], we next determined whether AKT/CCND1 axis altered the expression levels of E2F7 dependent of its effects on cellular proliferation and cell cycle. Our results showed that overexpression of AKT or CCND1 significantly enhanced the protein levels of E2F7 in HepG2 and Huh-7 cells transfected with AKT (Fig. 5i) or CCND1 (Fig. 5j) when compared to the corresponding control cells. Moreover, levels of intranuclear β-catenin were also enhanced in HepG2 and Huh-7 cells transfected with AKT (Fig. 5k) or CCND1 (Fig. 5l) when compared to the corresponding control cells.

### Expression and correlation of miRNA-302a/d and E2F7 in HCC

**Fig. 6:**
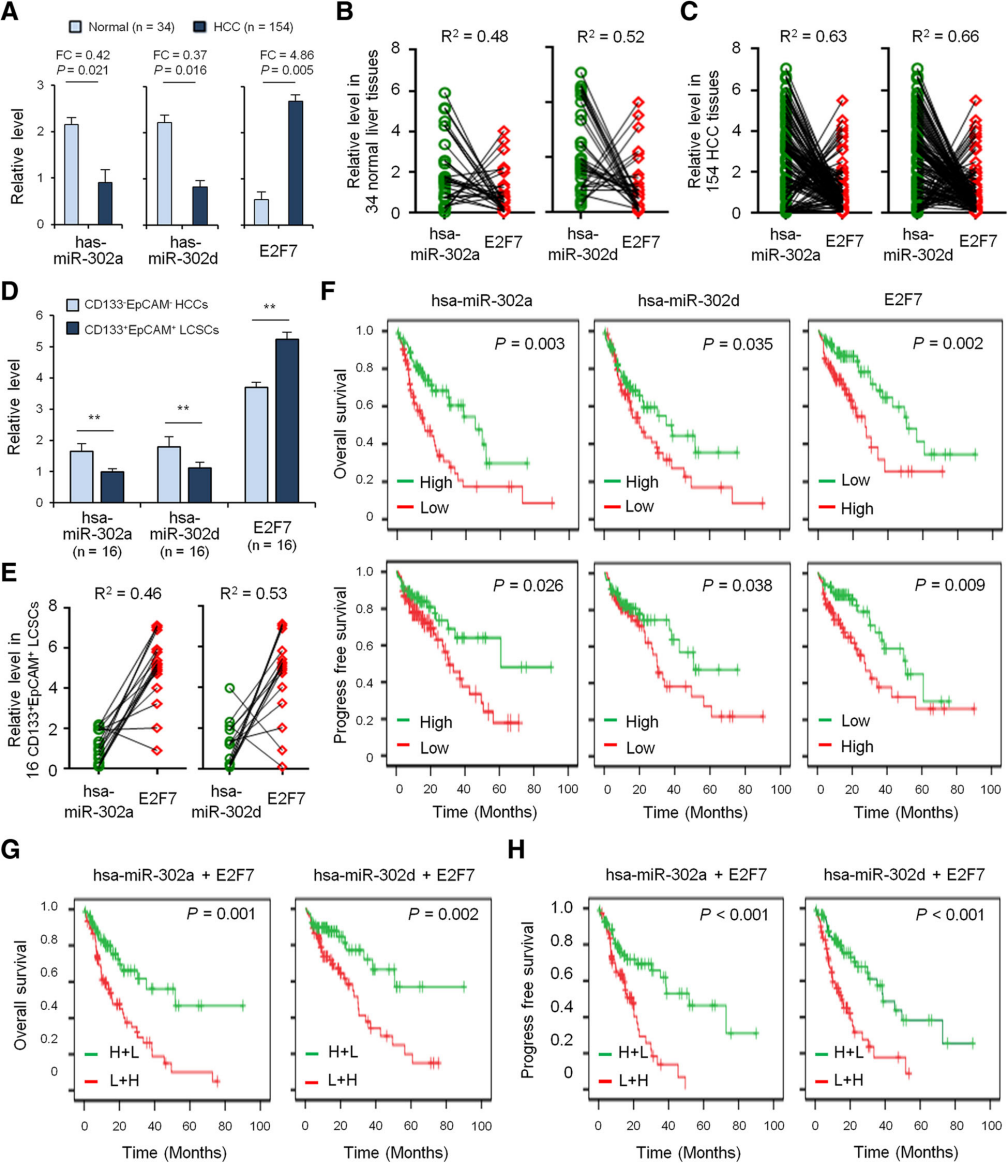
The expression levels and clinical significance of miRNA-302a/d and E2F7 in HCC. **a**, qRT-PCR to quantify miRNA-302a/d and E2F7 level in 154 HCC tissues and 34 adjacently normal liver tissues. The correlation between miRNA-302a/d and E2F7 levels in 34 adjacently normal liver tissues (**b**) and 154 HCC tissues (**c**). **d**, qRT-PCR to quantify miRNA-302a/d and E2F7 level in CD133^−^EpCAM^−^ HCCs and CD133^+^EpCAM^+^ LCSCs from 16 HCC tissues. **e**, The correlation between miRNA-302a/d and E2F7 levels in CD133^−^EpCAM^−^ HCCs and CD133^+^EpCAM^+^ LCSCs from 16 HCC tissues. **f**, Kaplan-Meier survival analysis to evaluate the prognostic value of miRNA-302a, miRNA-302d and E2F7 expression for OS and PFS of HCC patients. Kaplan-Meier survival analysis was used to evaluate the prognostic value of E2F7 together with miRNA-302a (**g**) or miRNA-302d (**h**) expression in HCC for OS and PFS

Next, we validated the expression levels of miRNA-302a/d and E2F7 in HCC tissue samples using qRT-PCR in 154 HCC biopsies, 34 of which were pairs of tissue from para-carcinoma tissues. MiRNA-302a level was downregulated in HCC tissues (FC = 0.42, *P* = 0.021) (Fig. 6a). When compared with normal liver tissues, the levels of miRNA-302d displayed a 0.37-fold decrease (*P* = 0.016) in 154 HCC biopsies (Fig. 6a). However, E2F7 expression analysis by qRT-PCR demonstrated that its expression levels were significantly higher in 154 HCC tumor biopsies relative to adjacent non-neoplastic tissues. This difference was statistically significant (FC = 4.86, *P* = 0.005, Fig. 6a).

Moreover, our results showed that E2F7 expression was negatively correlated with miRNA-302a/d expression in both normal liver tissues (*n* = 34, R^2^ = 0.48 and 0.52, respectively, Fig. 6b) and HCC tumor biopsies (*n* = 154, R^2^ = 0.63 and 0.66, respectively, Fig. 6c). The expression level of E2F7 was downregulated in the miRNA-302a/d-high expression samples, and upregulated in the miRNA-302a/d-low expression samples (Fig. 6b, c).

### Expression and correlation of miRNA-302a/d and E2F7 in LCSCs

Considering the close association of miRNA-302a/d with E2F7 in HCC cell lines and tissues, we next investigated the expression and correlation of miRNA-302a/d and E2F7 in LCSCs extracted from the human tissue specimen. Our results showed that miRNA-302a/d expression was significantly lower in CSC-enriched LCSCs compared with FACS-sorted CD133^−^EpCAM^−^ HCC cells (Fig. 6d). Moreover, a cluster of CD133^+^/EpCAM1^+^ hepatoma cells, which have been considered liver CSCs, exhibited enhanced E2F7 expression compared with the CD133^−^EpCAM^−^ cells (Fig. 6d). As expected, a negative correlation between miRNA-302a/d expression and the levels of E2F7 was observed in CD133^+^/EpCAM1^+^ hepatoma cells (Fig. 6e).

### Clinical significance of miRNA-302a/d and E2F7 in HCC

We then evaluated the prognostic value of miRNA-302a/d and E2F7 expression for patients with HCC. The overall survival (OS) and progression-free survival (PFS) in patients with a high expression of miRNA-302a or miRNA-302d was prolonged relative to patients with low expression of miRNA-302a or miRNA-302d (Fig. 6f). Next, we further explored the clinical significance of E2F7 in HCC patients. Our results indicated that E2F7 expression was positively correlated with lower OS (*P* = 0.002) and PFS (*P* = 0.009) (Fig. 6f) in HCC patients.

Since the data had suggested that E2F7 expression level was negatively correlated with miRNA-302a/d expression, and E2F7 was a direct target of miRNA-302a/d, we further examined the prognostic value of E2F7 expression together with miRNA-302a/d levels using multivariate analysis of OS and PFS. The results showed that HCC patients with high E2F7 expression and low miRNA-302a levels had significantly decreased OS (*P* = 0.001) and PFS (*P* = 0.002) (Fig. 6g). Similarly, HCC patients with high E2F7 expression and low miRNA-302d levels had significantly decreased OS (*P* < 0.001) and PFS (*P* < 0.001) (Fig. 6h), which suggested that E2F7 and miRNA-302a/d might have potential prognostic value and could be useful as tumor biomarkers for the diagnosis of HCC patients.

## Discussion

Recent experimental evidence has shown that CSCs show higher tumorigenic potential and resistance to chemo/radiotherapy compared to non-CSCs [32]. These features indicate that CSCs are involved in the development, progression, recurrence, and metastasis of tumors [33], suggesting that molecular and pathological characterization of CSCs is important to improve the prognoses of cancer patients. However, the precise roles and association between CSCs and the prognoses of patients are poorly understood, especially in HCC.

As regulators of diverse biological processes, different noncoding RNAs (ncRNAs) that negatively regulate gene expression, such as long ncRNAs (lncRNAs), are reported to be involved in the onset and development of HCC through LCSC regulation [34]**.** LncRNAs have the potential to serve as promising biomarkers for HCC progression and recurrence, as well as invasion and metastasis. Besides, lncRNAs are involved in HCC by affecting cell proliferation, apoptosis, and angiogenesis. Additionally, lncRNAs serve as a potential indicator of chemo-sensitivity and radio-resistance [35]. Meanwhile, lncRNAs exert their influence in interaction with proteins/miRNAs/mRNAs/lncRNAs via epigenetic regulation/transcriptional regulation, as well as in regulation of autophagy, signaling pathways, and mRNA stabilization in HCC. Therefore, lncRNAs might be promising indicators for disease diagnosis, prognosis and recurrence prediction and potentially up-and-coming targets for therapeutic intervention of HCC [36].

In addition, miRNAs, a large number of small noncoding RNA genes found to be aberrantly expressed in various types of malignancies that function as either oncogenes or tumor suppressors, are a sophisticated tool in therapeutics and diagnostics, and remain a prime focus among cancer researchers [37]. Several miRNAs, which are involved in stemness maintenance and differentiation, have been identified to play a critical role in regulating HCC tumorigenesis and drug resistance signaling networks [38]. Therefore, targeting LCSCs and elucidating the underlying mechanisms of miRNA in LCSCs may improve diagnostic and therapeutic strategies for HCC [39].

HCC is a primary liver tumor that causes high mortality and remains a global challenge with the current limited treatments available. Recent studies indicate that HCC arises from LCSCs leading to tumor heterogeneity [40]. Expression of some liver CSC markers such as CD133 and EpCAM has been reported to be associated with tumor angiogenesis, low response rates to chemotherapy, and poor overall prognoses in patients with HCC [41]. Moreover, HCC patients with high levels of circulating LCSCs were found to have higher recurrence rates than those with lower levels of CSCs [42]. Therefore, the expression of liver CSCs markers and their extraordinary characteristics can serve as potential prognostic markers and therapeutic target in these patients.

The miRNA-302 family was previously identified to be epigenetically silenced in various types of cancer and to play important roles in tumor development and progression [43]. The miRNA-302 family is significantly downregulated in human colorectal cancer, osteosarcoma, and glioblastoma tissues [44–46]. MiRNA-302 inhibits the tumorigenicity of endometrial cancer cells by suppressing cyclin D1 and CDK1 [47]. MicroRNA-302a was reported to suppress prostate tumor cell proliferation by inhibiting AKT, resulting in subsequent alterations of the AKT-GSK3β-cyclin D1 and AKT-p27 pathways [48]. Besides, overexpression of miR-302b suppresses HCC growth via targeting the EGFR/AKT2/CCND1 pathway [49]. These studies suggested that the miRNA-302 family plays an important role in tumorigenesis and progression. However, the clinical significance of the miRNA-302 family in HCC, and the molecular mechanisms underlying the deactivation of its target genes in tumorigenesis and drug resistance derived by LCSCs still require elucidation.

In this study, we used tumor sphere formation to enrich LCSC cells and found that the miRNA-302 family was weakly expressed in LCSC cells as well as in HCC tumors. Moreover, patients with lower miRNA-302a/d expression had shorter OS and PFS. We also identified that miRNA-302a/d inhibited HCC cell proliferation, tumor sphere formation in vitro and tumor growth in vivo. These results suggest that the miRNA-302 family is an important suppressor of LCSCs. Therefore, the potential anti-tumor mechanism of the miRNA-302 family in regulating LCSCs was further studied.

To uncover the underlying mechanism, four target gene prediction websites were used to forecast target genes of miRNA-302a/d and potential candidate and an atypical E2F family member E2F7, which limits the expression of E2F1 and prevents E2F1-dependent apoptosis [50–52], was selected and monitored in HepG2 and Huh7 cells with knocked down and overexpressed miRNA-302a/d. In this context, the expression of E2F7 was markedly inhibited by miRNA-302a/d. Owing to the typical seed sequence in the 3’-UTR of E2F7, a direct role of miRNA-302a/d was naturally surveyed next. As expected, E2F7 was a direct downstream target of miRNA-302a/d, as demonstrated by luciferase assays.

The members of the E2F family function as transcription factors and are widely expressed in a number of tissues and organs, they have been reported to control the expression of genes and possess many regulatory functions related to cellular proliferation, differentiation, DNA repair, the cell cycle, and cell apoptosis [53]. To date, eight members of this family, E2F1 to E2F8, have been recognized. In general, E2F1, E2F2, and E2F3 are considered to be transcriptional activators, whereas E2F4, E2F5, and E2F6 play inhibitory roles in transcriptional expression of downstream target genes [54]. The atypical E2F family member E2F8 and E2F7 perform complementary and overlapping functions in many cell metabolisms, and serve as a unique repressive arm to balance the E2F transcriptional network that is critical for embryonic development and control of the E2F1-p53 apoptotic axis [55]. E2F8 was found to be markedly overexpressed in HCC to facilitate tumor occurrence and development via activation of an E2F1/cyclin D1 signaling pathway to regulate the G1- to S-phase transition of cell cycle progression or transcriptionally suppress CDK1 to induce hepatocyte polyploidization [56]. Moreover, the miR-302-367 cluster was reported to directly downregulate both AKT1 and cyclin D1, and indirectly upregulated p27^Kip1^ and p21^Cip1^, leading to the suppression of cancer cell proliferation [47], which is accordance with our data (Fig. 4).

In this study, E2F7 was found to be overexpressed in LCSCs and HCC tissue and had an inverse relationship with miRNA-302a/d. Our results further implied that E2F7 could be modulated by miRNA-302a/d during HCC cells proliferation and apoptosis driven by LCSCs. More interestingly, E2F7 overexpression could activate AKT1-cyclin D1 signaling and the downstream cell cycle, and the levels of intranuclear β-catenin were strikingly enhanced.

Moreover, the feedforward AKT/CCND1 axis regulates E2F7 signaling dependent on its effects on cellular proliferation and the cell cycle, which results in positive E2F7/AKT/β-catenin/CCND1 feedback signaling to promote the self-renewal ability and cell cycle entry of LCSCs to act as enhancers for cell cycle progression and hepatocarcinogenesis.

To further evaluate their clinical signatures, miRNA-302a/d and E2F7 were divided into high- and low-expressing groups in HCC patients. In this context, E2F7 was highly expressed in HCC tissues when compared with normal liver tissues, and high levels of E2F7 in tumor tissues had poor prognostic values on the OS of HCC patients. Furthermore, concomitant low expression of miRNA-302a/d and high expression of E2F7 correlated with shorter median OS and PFS in HCC patients, suggesting miRNA-302a/d is a key regulator of HCC stemness via targeting the E2F7.

## Conclusions

Taken together, our findings indicate that miRNA-302a/d can inhibit the stemness and proliferation of HCC cells through targeting E2F7 and downstream AKT/β-catenin/CCND1 signaling and may play an essential role in HCC progression suggesting that miRNA-302a/d and E2F7 may be promising therapeutic targets for HCC patients.

## Additional file

Additional file 1: **Table S1.** miR-302a/d and E2F7 expression and clinicopathological characteristics of 154 HCC patients. **Table S2.** Realtime PCR primers used in this study. **Figure S1.** Expression of CSC Markers and miRNA-302 family in LCSCs. **Figure S2.** Expression of CSC Markers and miRNA-302 family during LCSCs differentiation. **Figure S3.** Validation of the correlation of miRNA-302a/d and E2F7 level. **Figure S4.** Biological role of miRNA-302a/d and E2F7 in HCC in vitro. (DOCX 1899 kb)

## Abbreviations

AJCC: American Joint Commission on Cancer
DFS: Disease-free survival
E2F7: E2F transcription factor 7
HCC: hepatocellular carcinoma
KEGG: Kyoto Encyclopedia of Genes and Genomes
LCSCs: Liver cancer stem cells
miRNA: MicroRNA
OS: Overall survival
qPCR: Quantitative PCR
RIP: RNA immunoprecipitation
TNM: Tumor-node-metastasis
