# Supplementary material for: MicroRNA-302a/d inhibits the self-renewal capability and cell cycle entry of liver cancer stem cells by targeting the E2F7/AKT axis
Source: J Exp Clin Cancer Res. 2018 Oct 16;37:252. doi: 10.1186/s13046-018-0927-8 (PMC6192354; doi:10.1186/s13046-018-0927-8)
Supplement: Supplementary file 1 — Table S1. miR-302a/d and E2F7 expression and clinicopathological characteristics of 154 HCC patients. Table S2. Realtime PCR primers used in this study. Figure S1. Expression of CSC Markers and miRNA-302 family in LCSCs. Figure S2. Expression of CSC Markers and miRNA-302 family during LCSCs differentiation. Figure S3. Validation of the correlation of miRNA-302a/d and E2F7 level. Figure S4. Biological role of miRNA-302a/d and E2F7 in HCC in vitro. (DOCX 1899 kb) [file 13046_2018_927_MOESM1_ESM.docx]

| **Table S1. miR-302a/d and E2F7 expression and clinicopathological characteristics of 154 HCC patients** | | | | | | | | | | | | | | |
| --- | --- | --- | --- | --- | --- | --- | --- | --- | --- | --- | --- | --- | --- | --- |
| Factor | Variable | Number | Overall survival | | | miR-302a expression | | | miR-302d expression | | | E2F7 expression | | |
|  |  |  | Months (Mean) | 95% CI (Mean) | *P* value | Low | High | *P* value | Low | High | *P* value | Low | High | *P* value |
| Age | ≥ 60 | 86 | 40.36 | 36.45 - 46.37 | 0.446 | 40 | 46 | 0.417 | 42 | 44 | 0.871 | 48 | 38 | 0.166 |
|  | < 60 | 68 | 47.12 | 42.58 - 51.64 |  | 37 | 31 |  | 35 | 33 |  | 29 | 39 |  |
| Gender | Male | 109 | 41.27 | 36.05 - 49.43 | 0.125 | 58 | 51 | 0.288 | 59 | 50 | 0.156 | 50 | 59 | 0.156 |
|  | Female | 45 | 43.33 | 38.54 - 51.02 |  | 19 | 26 |  | 18 | 27 |  | 27 | 18 |  |
| Tumor differentiation | Poorly | 89 | 21.59 | 15.79 - 30.26 | 0.008 | 54 | 35 | 0.003 | 53 | 36 | 0.002 | 37 | 52 | 0.022 |
|  | Good | 65 | 40.38 | 34.58 - 48.74 |  | 23 | 42 |  | 20 | 45 |  | 40 | 25 |  |
| Diameter | ≥ 5 cm | 95 | 36.89 | 29.55 - 41.26 | 0.019 | 56 | 39 | 0.008 | 56 | 39 | 0.008 | 37 | 58 | 0.001 |
|  | < 5 cm | 59 | 46.13 | 39.93 - 51.25 |  | 21 | 38 |  | 21 | 38 |  | 40 | 19 |  |
| Number of foci | Multiple | 81 | 34.57 | 28.42 - 41.55 | 0.012 | 50 | 31 | 0.004 | 48 | 33 | 0.024 | 32 | 49 | 0.009 |
|  | Single | 73 | 48.95 | 40.18 - 53.47 |  | 27 | 46 |  | 29 | 44 |  | 45 | 28 |  |

| **Table S2. Realtime PCR primers used in this study.** | |
| --- | --- |
| Primers | Sequences |
| CD133 (Forward) | 5'-AGTCGGAAACTGGCAGATAGC-3' |
| CD133 (Reverse) | 5'-GGTAGTGTTGTACTGGGCCAAT-3' |
| EpCAM (Forward) | 5'-GGCGTGTGGAGATGTATAAC-3 |
| EpCAM (Reverse) | 5'-CAGCTCAGCACTATCCAGAC-3' |
| Sox2 (Forward) | 5'-GTCATTTGCTGTGGGTGATG-3' |
| Sox2 (Reverse) | 5'-AGAAAAACGAGGGAAATGGG-3' |
| Nanog (Forward) | 5'-AATACCTCAGCCTCCAGCAGATG-3' |
| Nanog (Reverse) | 5'-TGCGTCACACCATTGCTATTCTTC-3' |
| E2F7 (Forward) | 5'-TGTGAGCTATCTGGAAGAACC-3' |
| E2F7 (Reverse) | 5'-TTCAGTCCGACTGGTCACTCA-3' |
| GAPDH (Forward) | 5'-ACCCAGAAGACTGTGGATGG-3' |
| GAPDH (Reverse) | 5'-TTCTAGACGGCAGGTCAGGT-3' |

**Figure Legends**

**
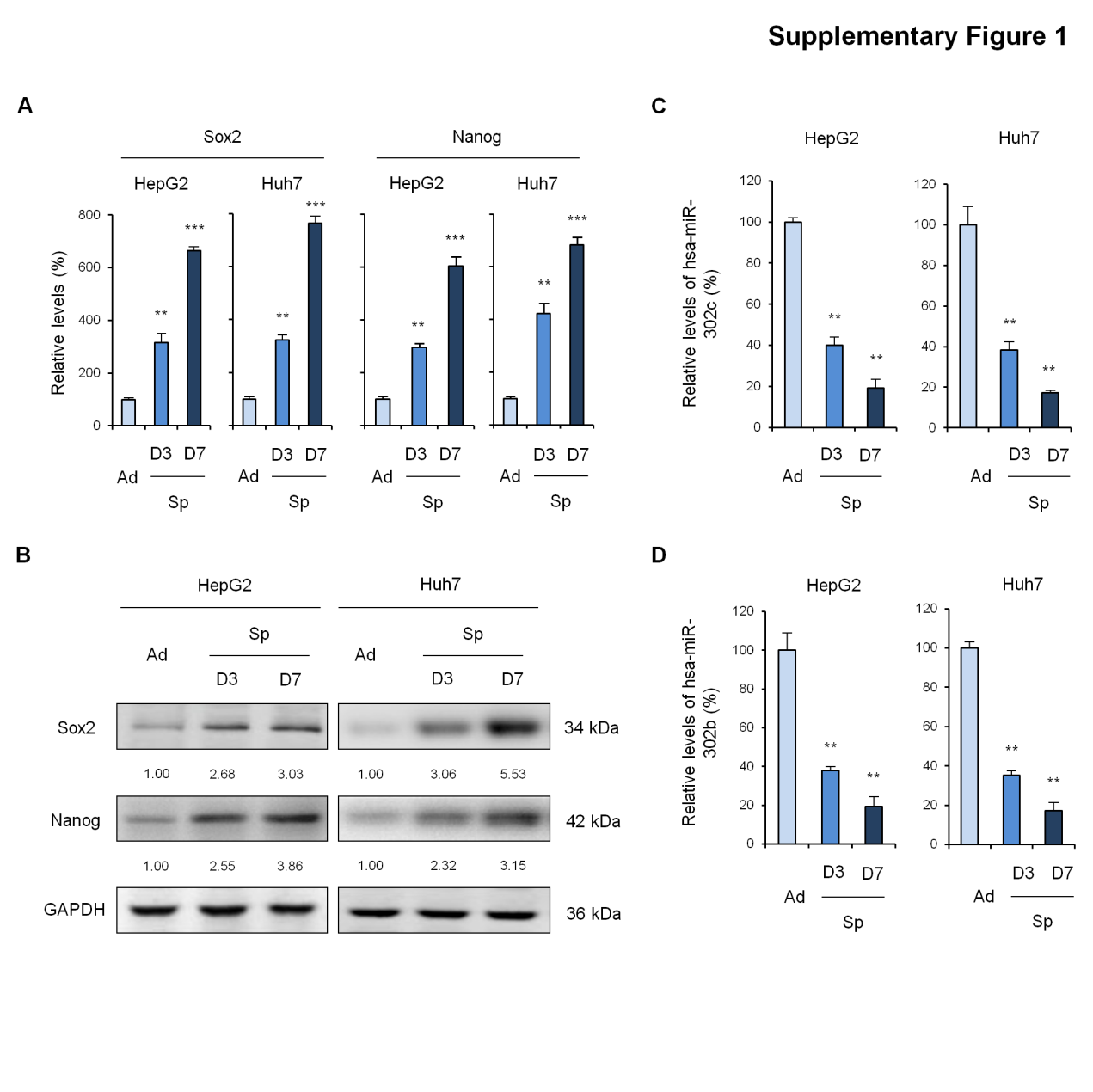
Figure S1****:** **Expression of CSC Markers and miRNA-302 family in LCSCs.** qRT-PCR (A) and Western blot (B) to quantify embryo stem cell markers Sox2 and Nanog in adherent and tumor spheres of HepG2 and Huh7 cells after 3 and 7 days cultured in stem cell medium containing EGF and bFGF. qRT-PCR to validate the miRNA-302c (C) and -302b (D) level in adherent and tumor spheres of HepG2 and Huh7 cells after 3 and 7 days cultured in stem cell medium containing EGF and bFGF. Data shown are the means ± SD of three independent experiments. Statistical analyses were performed with one-way ANOVA (*P<0.05, **P<0.01, and ***P<0.001 vs. normal).

**
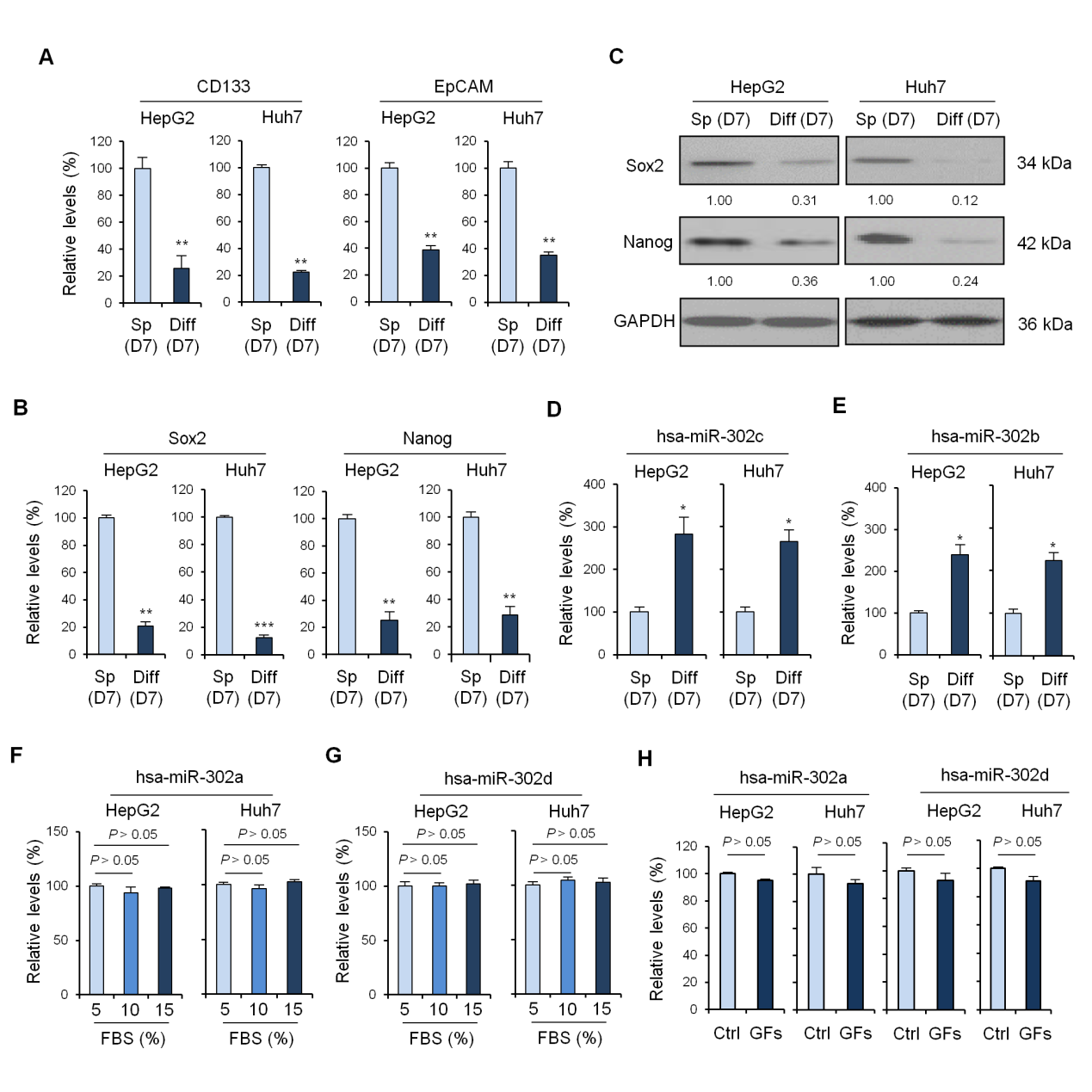
**

**Figure S2: Expression of CSC Markers and miRNA-302 family during LCSCs differentiation.** qRT-PCR to quantify CD133 and EpCAM level in tumor spheres of HepG2 and Huh7 cells at Day 7 cultured in stem cell medium and differentiated lung cancer cells. qRT-PCR (B) and Western blot (C) to quantify embryo stem cell markers (Sox2 and Nanog) in tumor spheres of HepG2 and Huh7 cells at Day 7 cultured in stem cell medium and differentiated lung cancer cells. qRT-PCR to validate the miRNA-302c (D) and -302b (E) in adherent and tumor spheres of HepG2 and Huh7 cells at Day 7 cultured in stem cell medium and differentiated lung cancer cells. qRT-PCR to validate the effect of different concentration of FBS on mRNA levels of miRNA-302a (F) and -302d (G). H, qRT-PCR to validate the effect of additional growth factors (containing 20 ng/mL hEGF, 10 ng/mL hbFGF, 4 mg/mL heparin sulfate) on mRNA levels of miRNA-302a and -302d. Data shown are the means ± SD of three independent experiments. Statistical analyses were performed with one-way ANOVA (^#^P>0.05, *P<0.05, **P<0.01, and ***P<0.001 vs. normal).

**
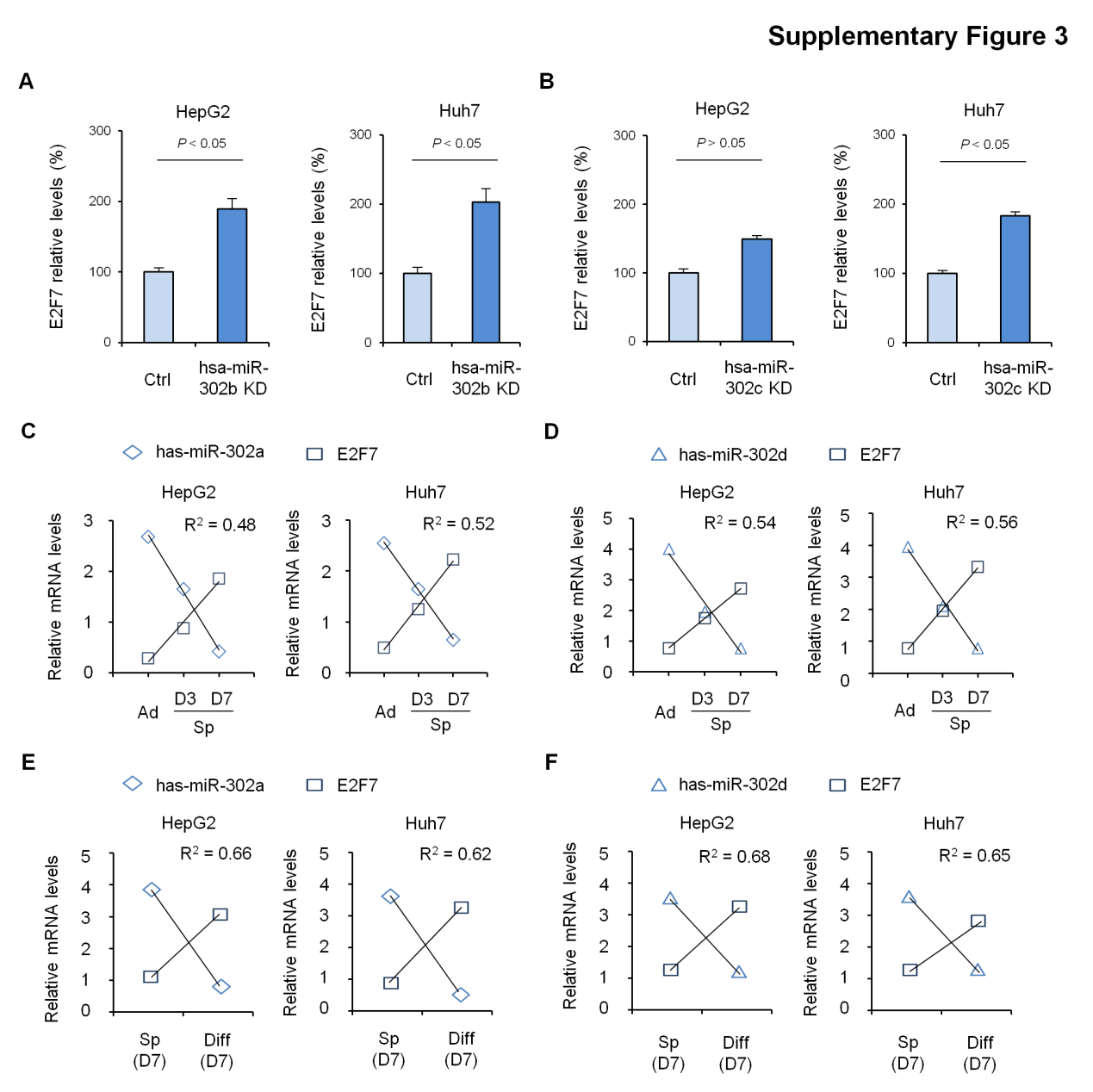
Figure S3:** **Validation of the correlation of miRNA-302a/d and E2F7 level.** qRT-PCR were used to measure the mRNA level of E2F7 after treatment of miRNA-302b (A) or -302d (B) knockdown. A. The correlation of miRNA-302a (C) and -302d (D) with E2F7 level in adherent and tumor spheres of HepG2 and Huh7 cells after 3 and 7 days cultured in stem cell medium containing EGF and bFGF. The correlation of miRNA-302a (E) and -302d (F) with E2F7 level in tumor spheres of HepG2 and Huh7 cells at Day 7 cultured in stem cell medium and differentiated lung cancer cells.

**
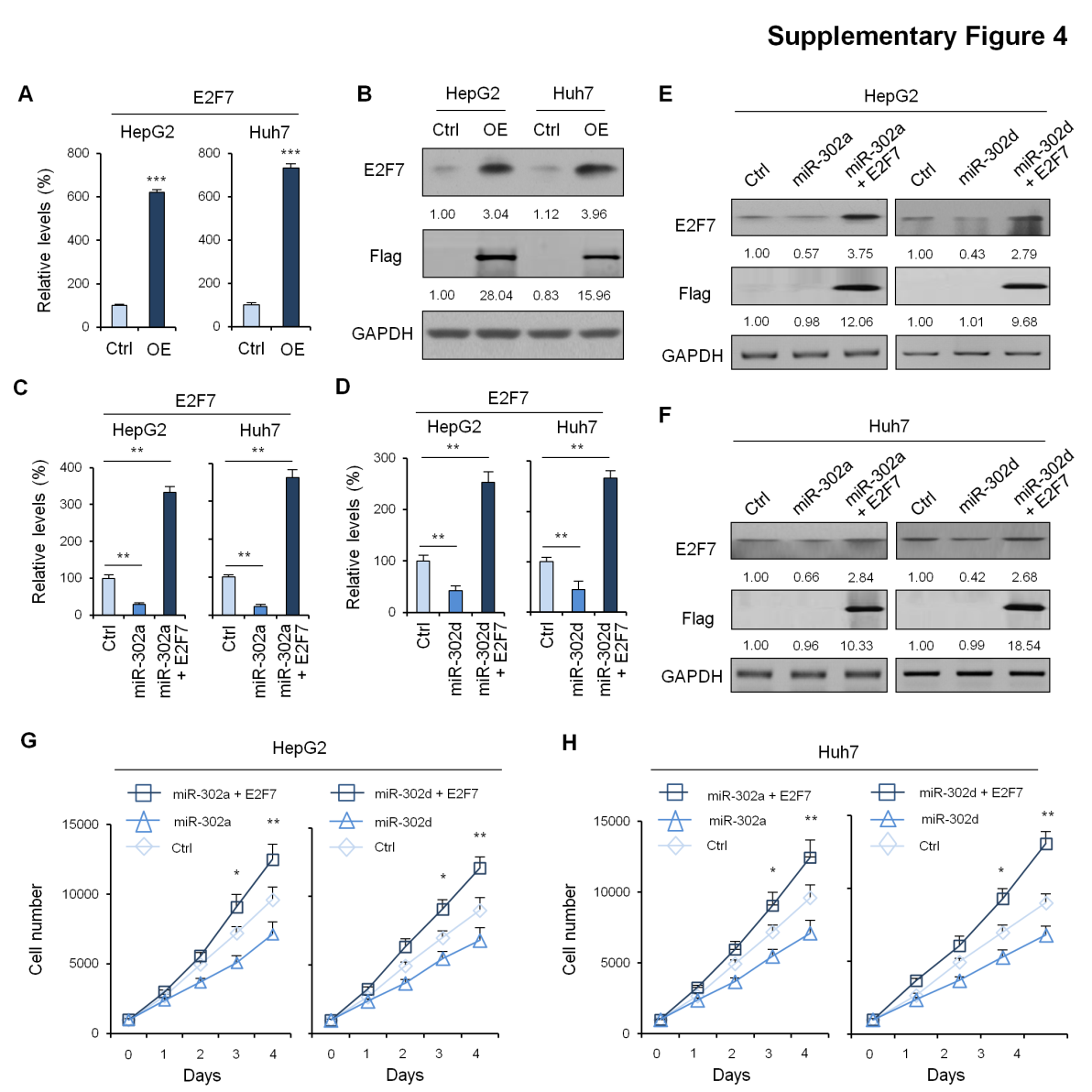
Figure S4: Biological role of miRNA-302a/d and E2F7 in HCC *in vitro*.** qRT-PCR (A) and Western blot (B) measurement of the levels of E2F7 in HepG2 and Huh7 cells before and after overexpression of E2F7. qRT-PCR measurement of the levels of E2F7 in HepG2 and Huh7 cells before and after overexpression of miRNA-302a (C) and -302d (D) and/or E2F7. Western blot measurement of the protein levels of E2F7 and Flag in HepG2 and Huh7 cells before and after overexpression of miRNA-302aand -302d (F) and/or E2F7. HepG2 (G) and Huh7 (H) cell counts in 96-well plate after transfection with negative control and miRNA-302a/d and/or E2F7 overexpression plasmids at the indicated day.
